# A comparison of patient characteristics, prognosis, treatment modalities, and survival according to age group in gastric cancer patients

**DOI:** 10.1186/1477-7819-10-234

**Published:** 2012-11-02

**Authors:** Deniz Tural, Fatih Selçukbiricik, Süheyla Serdengeçti, Evin Büyükünal

**Affiliations:** 1 Department of Internal Medicine, Division of Medical Oncology, Cerrahpasa Medical School, Istanbul University, Istanbul, 34098, Turkey

**Keywords:** Elderly, Gastric cancer, Characteristics, Population, Prognosis, Treatment, Outcome

## Abstract

**Background:**

The aim of this study was to investigate age-specific incidence rates and to compare disease stage, treatment, and survival according to age group in patients with gastric adenocarcinoma.

**Methods:**

Gastric cancer patients treated at our hospital between 1999 and 2010 were retrospectively evaluated. We divided the cases into two subgroups: group 1 consisted of patients older than 70 years at the time of treatment, and group 2 included patients aged 70 years or younger. In all, 151 patients over 70 years of age and 715 patients age 70 years or younger were analyzed. Categorical and continuous variables were summarized using descriptive statistics and compared using statistical software. Overall survival rates were estimated via the Kaplan-Meier method.

**Results:**

Median age at diagnosis was 58 years (range: 22 to 90 years). Between 1999 and 2002 the annual median age for patients aged older than70 years was 9.8%, which increased to 20% between 2007 and 2010. The one-year survival rate for patients with metastatic disease (stage IV) was 10.9% (95% CI: 8.9% to 12.9%) and 27.8% (95% CI: 17.3% to 38.2%) in groups 1 and 2, respectively (*P* = 0.015). The five-year survival rate for patients with non-metastatic disease (in whom curative surgery was performed) was 15.5% (95% CI = 12% to 19%) and 26.9% (95% CI = 25.9% to 27.9%) in groups 1 and 2, respectively (*P* = 0.03). There were no significant differences in gender, tumor localization in the stomach, tumor histology, perineural invasion (PNI), lymphovascular invasion (LVI), tumor stage, or type of surgery between the two groups. However, fewer of the patients in group 1 underwent adjuvant treatment (*P* = 0.02) and palliative chemotherapy (*P* = 0.007) than group 2 patients that were non-metastatic and metastatic at presentation, respectively.

**Conclusions:**

Groups 1 and 2 were similar in terms of histopathological features and surgical modality; however, the survival rate was lower in group 1 than in group 2. The incidence of gastric cancer was higher in the patients older than 70 years of age. Additional randomized studies are needed to further assess the safety and clinical benefit of chemotherapy in gastric cancer patients older than70 years of age.

## Background

The incidence and mortality rates for most cancers are decreasing in the United States and in other developed Western countries [1,2]; however, cancer still accounts for more deaths than heart disease in those aged 85 years and younger [3]. Cancer in older people has become an increasingly common problem due to the prolonged life expectancy of the general population.

The overall gastric cancer incidence and mortality rates are decreasing worldwide, but despite the recent decline, gastric cancer remains the fourth most common cancer and the second leading cause of cancer-related mortality [4-6]. Moreover, the incidence of gastric cancers increases with age, especially in the United States, and most gastric cancer patients in Japan are older [7,8]. The age-adjusted mortality rate due to gastric cancer was reported to increase with age [9].

The aim of the present study was to use data collected from 886 patients in our hospital-based registry who were treated between 1999 and 2010 to evaluate age-specific incidence rates, and to compare histopathology, disease stage, treatment modalities, and outcomes according to age group in patients with gastric adenocarcinoma.

## Methods

The study included gastric cancer patients treated at Istanbul University’s Cerrahpaşa Medical School between 1999 and 2010. The cancer in each patient was coded according to the *International Classification of Diseases for Oncology*[10]. The cases were divided into two subgroups based on age: group 1 consisted of patients older than 70 years of age at the time of treatment, and group 2 was composed of patients aged 70 years or younger. The patients were further categorized into five age groups: younger than 40 years, 40 to 50 years, 50 to 60 years, 60 to 69 years, and 70 years and older. Groups 1 and 2 were compared in terms of tumor localization in the stomach, histopathological subtype, age at diagnosis, disease stage, neuronal invasion, lymphovascular invasion (LVI), and treatment modality.

The cases were also divided into the following two subgroups based on anatomic localization of cancer in the stomach: cardia cancer subgroup and non-cardia cancer subgroup (gastric antrum, pylorus, lesser and greater curvature of the stomach) [11]. In addition, the cases were divided into three subgroups based on histopathological subtype, as follows: subtype subgroup 1 included patients with intestinal gastric cancer, subtype subgroup 2 consisted of those with diffuse gastric cancer, and subtype subgroup 3 was mixed [12]. Disease stage was determined according to the seventh edition of the International Union Against Cancer classification system. Stage I, II, and III were classified as non-metastatic and stage IV was classified as metastatic. Total gastrectomy, or subtotal gastrectomy and lymph node dissection was performed in all patients with non-metastatic presentation.

In non-metastatic patients the protracted 5-fluorouracil (5FU) infusion chemotherapy regimen was used concurrently with postoperative radiotherapy (RT). Five cycles of adjuvant bolus 5FU (425 mg·m^-2^·d^-1^) and leukoverin (20 mg·m^-2^·d^-1^) as a Mayo regimen was administered on treatment day 1 to day 5 every 28 days to these patients after surgery. Bolus 5FU and leukoverin were administered on treatment day 1 to day 4 every 28 days concurrently with postoperative RT during the 2nd and 3rd cycles of the planned five-cycle adjuvant chemotherapy. Radiotherapy (RT) was administered (range: 45 to 50.4 Gy) to the tumor bed and draining lymph nodes in cases with 2- to 3-cm margins.

Patients with positive surgical margins, incomplete chemoradiotherapy(CRT), poor performance status (Eastern Cooperative Oncology Group (ECOG) >2), inadequate renal and hepatic function, and other second primary cancers were excluded from the study. Adjuvant chemotherapy was administered to stage IB, II, and III patients, and palliative chemotherapy was administered to stage IV patients. 40.1% of patients received docetaxel (75 mg/m^2^) on day 1 and cisplatinum (75 mg/m^2^) the next day intravenously plus infusion 5FU (750 mg/m^2^) continuously on days 1 to 5 (TCF), 30% of patients received epirubicin (50 mg/m^2^), cisplatin (60 mg/m^2^) and 5FU (200 mg/m^2^) infusion (ECF), 20.2% of patients received bolus 5FU (425 mg/m^2^) plus leucovorin (20 mg/m^2^) on days 1 to 5 (FUFA) and 9.7% of patients received other chemotherapy regimens for palliation in the metastatic stage.

### Statistical analysis

Categorical and continuous variables were summarized using descriptive statistics (e.g., median, range, frequency, and percentage) and compared using the chi-square and Mann-Whitney U tests, respectively. The median age rates between 1999 and 2010 for the five age subgroups were calculated for one-year and four-year periods. Overall survival rates (OS) and disease-free survival (DFS) were estimated via the Kaplan-Meier method. OS was calculated as the time from the date of diagnosis to the time of death due to any cause, and DFS was calculated as the time from the date of diagnosis to the time of recurrence. Any differences in survival between categorical variables were tested using the log-rank test. Based on the results of univariate analysis, we performed multivariate analysis using a Cox proportional hazard model to calculate the hazard ratio (HR) and 95% confidence interval (CI). All analyses were performed using SPSS v.15.0 for Windows (SPSS Inc., Chicago, Illinois, USA) software. The level of statistical significance was set at *P* <0.05.

## Results

**Figure 1:**
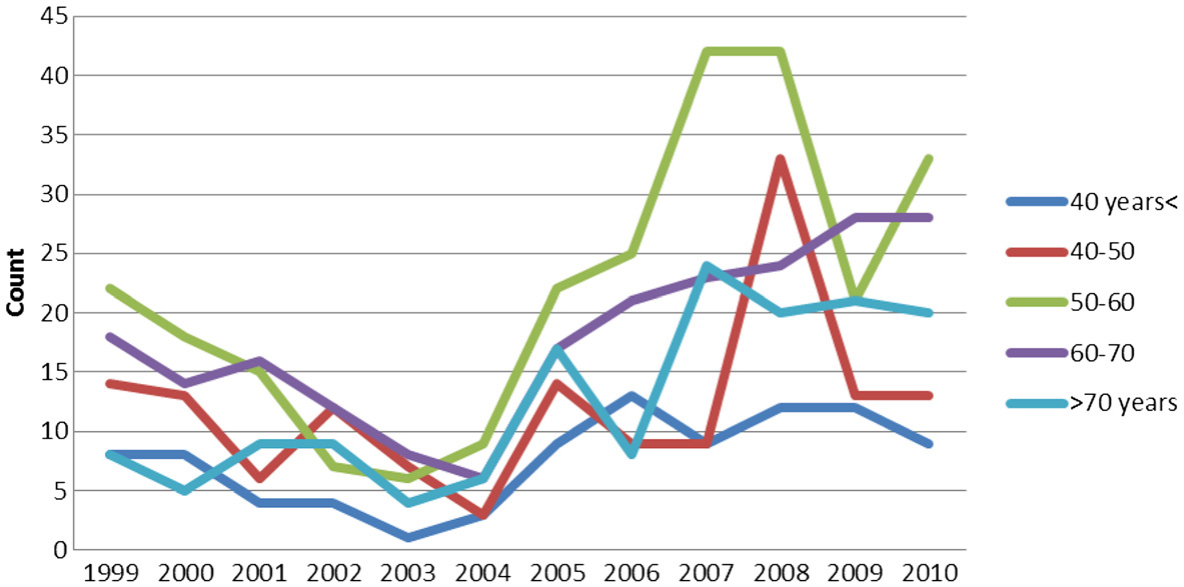
Changes in the annual median age of gastric adenocarcinoma patients shown in one-year periods between 1999 and 2010.

The hospital-based registry included 866 cases of gastric adenocarcinoma that were treated between 1999 and 2010. Median age at diagnosis was 58 years (range: 22 to 90 years) (57 years between 1999 and 2002, 58 years between 2003 and 2006, and 59 years between 2007 and 2010). The ratio of patients aged 60 to 70 years and older than 70 years increased from 1999 to 2010; therefore, advanced age was strongly correlated with the incidence of gastric adenocarcinoma (*P* = 0.02) (Figure 1).

**Figure 2:**
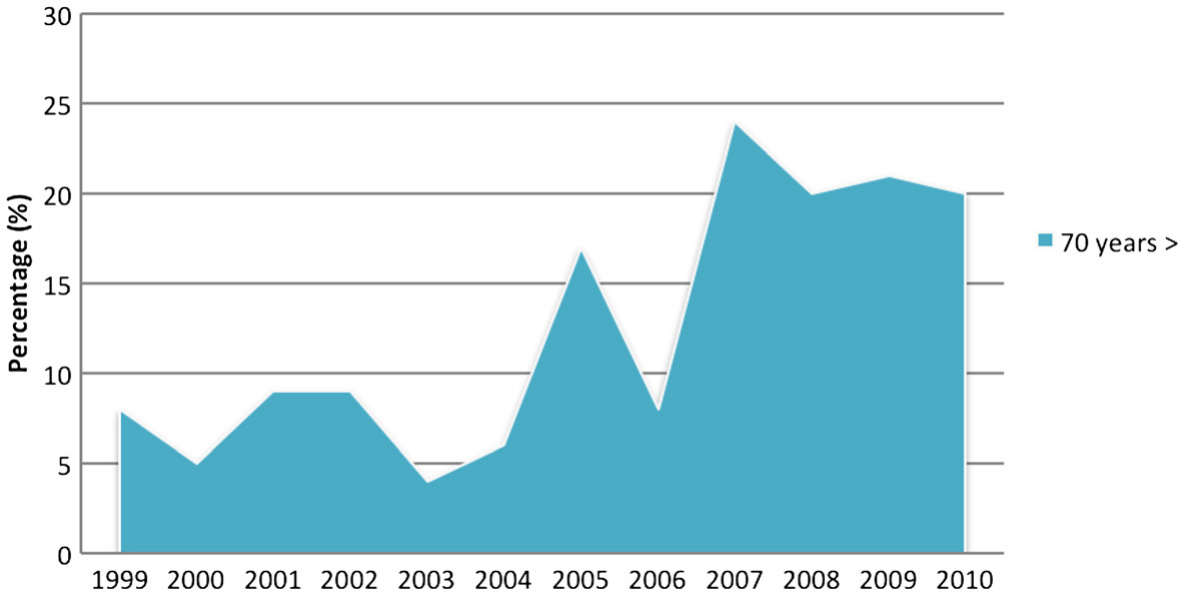
Changes in the annual values rates for patients older than70 years of age are shown in one-year periods between 1999 and 2010.

Between 1999 and 2002 the annual median age for patients older than 70 years was 9.8%, which increased to 20% between 2007 and 2010 (*P* = 0.001) (Figure 2).

There were no significant differences in gender, tumor localization in the stomach (cardia/non-cardia), tumor histology, perineural invasion (PNI), lymphovascular invasion (LVI), tumor stage, or type of surgery between groups 1 and 2; however, fewer of the patients in group 1 underwent adjuvant treatment (*P* = 0.02) and palliative chemotherapy (*P* = 0.007) than group 2 patients that were non-metastatic and metastatic at presentation, respectively. The correlation between age, and clinicopathological factors and treatment modalities is shown in Table 1.

**Table 1:** Clinicopathological features and treatment modalities in groups 1 and 2

**Variable**	**Group 2 (age**≤**70 years ) n (%) Group 1 (age >70 years) n (%)**	***P p***
**Gender**		**0.26**
Male	504 (70.5)	
	102 (67.5)	
Female	211 (29.5)	
	49 (32.5	
**ECCOG**		**0.14**
0	41 (27)	
	210 (29)	
1	80 (53)	
2	405 (56)	
	30 (20)	
	100 (15)	
**Histology**		**0.65**
Diffuse adenocarcinoma	229 (32)	
	36 (24)	
Intestinal adenocarcinoma	437 (61)	
	99 (66)	
Mixed adenocarcinoma	49 (7)	
	16 (10)	
**Stomach localization**		**0.2**
Proximal (cardia)	127 (17.8)	
	31 (19.6)	
Distal part	588 (82.2)	
	120 (79.5)	
**Presentation type**		**0.4**
Metastatic	345 (51.6)	
	68 (52.3)	
Non-metastatic	324 (48.4)	
	62 (47.7)	
**Perineural invasion**		**0.5**
Present	210 (73)	
Absent	40 (74)	
	77 (27)	
	14 (26)	
**Lymphovascular invasion**		**0.21**
Present	230 (80.5)	
Absent	56 (19.5)	
	48 (80)	
	12 (20)	
**Surgical procedure**		**0.5**
Subtotal gastrectomy	184 (56.8)	
	30 (48.4)	
Total gastrectomy	133 (41)	
	30 (48.4)	
Palliative surgery	7 (2.2)	
	2 (3.2)	
**Adjuvant treatment**		**0.02**
Present	244 (75)	
	37 (60)	
Absent	65 (20)	
	19 (30)	
Unknown	15 (5)	
	6 (10)	
**Palliative Treatment**		**0.007**
Chemotherapy	287 (83.2)	
	46 (67.6)	
Palliative care	45 (13)	
	15 (22.1)	
Unknown	13 (3.8)	
	7 (10.3)	

**Figure 3:**
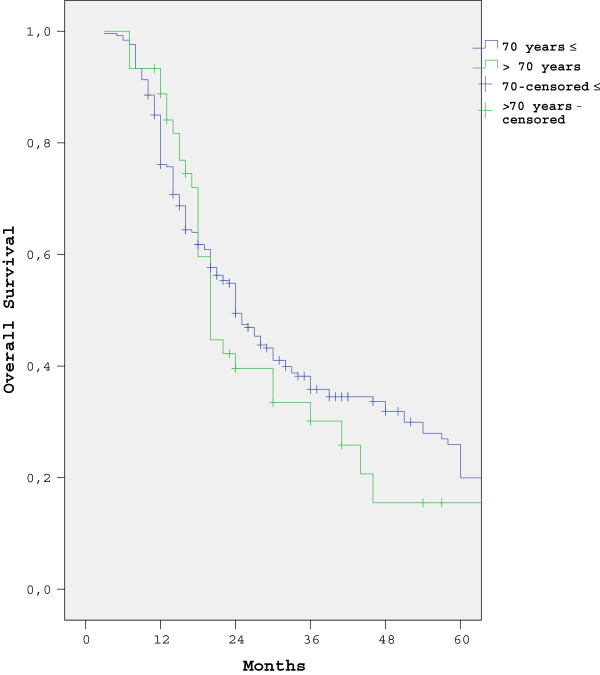
Five-year survival rates according to age group in patients with non-metastatic presentation.

**Figure 4:**
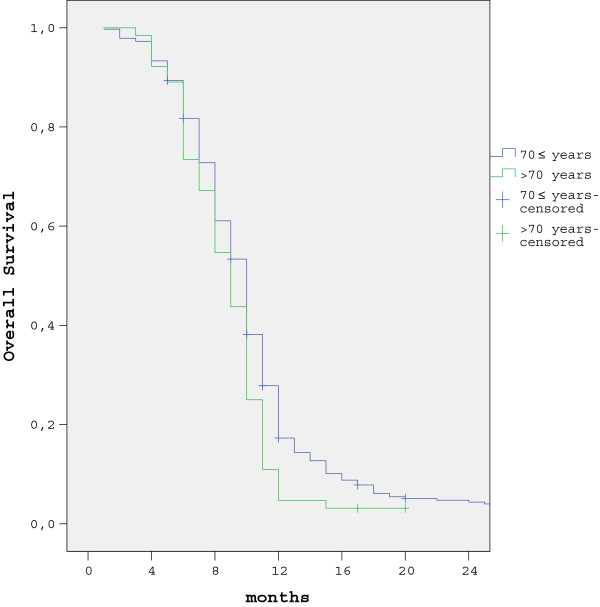
One-year survival rates according to age group in patients with metastatic presentation.

The five-year survival rate in patients with non-metastatic presentation was 15.5% (95% CI = 12% to 19%) and 26.9% (95% CI = 25.9% to 27.9%) in groups 1 and 2, respectively (*P* = 0.03). The one-year survival rate in metastatic gastric cancer patients was 10.9% (95% CI = 8.9% to 12.9%) and 27.8% (95% CI = 17.3% to 38.2%) in groups 1 and 2, respectively (*P* = 0.015). The survival curves for both groups are shown in Figures 3 and 4.

In non-metastatic patients (n = 386) univariate analysis was conducted based on age subgroup, gender, PNI, LVI, disease stage (I, II, and III), histology subtype, tumor localization in the stomach, and surgical method, along with whether or not the patient received adjuvant treatment. Early-stage cancer (stage I and II) (*P* = 0.001), absence of PNI (*P* = 0.004), absence of LVI (*P* = 0.007), adjuvant treatment (*P* = 0.04), and age 70 years or younger (*P* = 0.03) were associated with better survival rates. In metastatic patients (413) we conducted univariate analysis based on age subgroup, gender, histological subtype, and palliative treatment modality, and observed that being age 70 years or younger (*P* = 0.015) and undergoing palliative chemotherapy (*P* = 0.001) were associated with better survival rates. Based on these results, we performed multivariate analysis using a Cox proportional hazard model. Among the non-metastatic gastric cancer patients, those who received adjuvant treatment had a HR of 0.4 (95% CI: 0.26 to 0.6), those with PNI had an HR of 1.67 (95% CI: 1.08 to 2.78), those with LVI had an HR of 2.8 (95% CI: 1.2 to 6.6), and those with stage III cancer had an HR of 3.0 (95% CI: 1.57 to 5.87). Among the patients with metastatic gastric cancer, those who received palliative chemotherapy, as opposed to palliative care, had an HR of 0.58 (95% CI: 0.44 to 0.77) and those older than 70 years had an HR of 1.3 (95% CI: 1.02 to 1.7). The details of Cox regression analysis are shown in Tables 2 and 3.

**Table 2:** Results of Cox regression analysis of the association between overall survival rates, and perineural invasion, lymphovascular invasion, and disease stage in 386 patients with non-metastatic presentation

**Variable**	***P***	**Overall survival**
		HR (95% CI)
**Adjuvant Treatment**	0.01	
No		1
Yes		0.4 (0.26 to 0.6)
**Perineural invasion**	0.043	
Absent		1
Present		1.67 (1.08 to 2.78)
**Lymphovascular invasion**	0.021	
Absent		1
Present		2.8 (1.2 to 6.6)
**Stage**	0.001	
Stage I		1
Stage II		1.4 (1.09 to 1.71)
Stage III		3 (1.57 to 5.87)
≤70 years	0.03	1
>70 years		1.27(1.17 to 1.48)

CI, confidence interval; HR; hazard ratio.

**Table 3:** Results of Cox regression analysis of the association between overall survival rates, and palliative treatment and age at diagnosis in 413 patients with metastatic presentation

**Variable**	***P***	**Overall survival**
		HR (95% CI)
**Palliative Treatment**	0.001	
Palliative care		1
Palliative chemotherapy		0.58 (0.44 to 0.77)
**Age at diagnosis**	0.04	
≤70 years		1
>70 years		1.3 (1.02 to 1.7)

CI, confidence interval; HR, hazard ratio.

**Figure 5:**
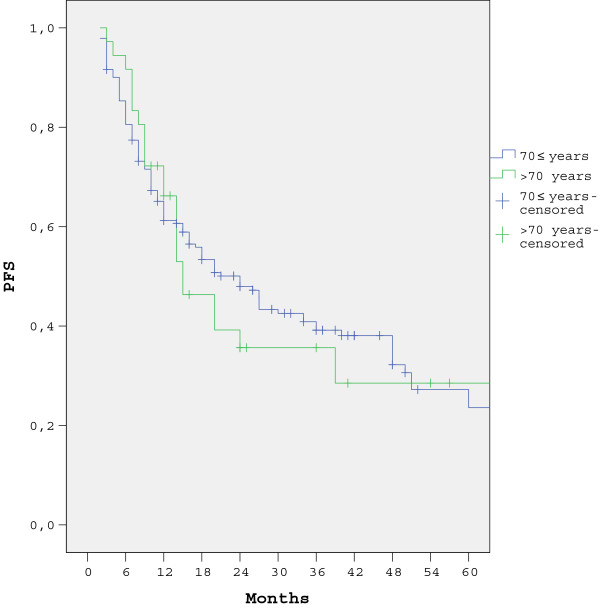
Five-year PFS rates according to age group in patients with non-metastatic presentation.

The five-year DFS rate in patients with non-metastatic presentation was 40.1% (95% CI = 39.9% to 42.5%) and 35.6% (95% CI = 32.9% to .41.1%) in groups 1 and 2, respectively (*P* = 0.59). Univariate analysis was conducted based on age subgroup, gender, PNI, LVI, disease stage (I, II, and III), histology subtype, tumor localization in the stomach, and surgical method, along with whether or not the patient received adjuvant treatment. Early-stage cancer (stage I and II) (*P* = 0.02), absence of PNI (*P* = 0.03), absence of LVI (*P* = 0.006), and adjuvant treatment (*P* = 0.03), were associated with better DFS rates. The DFS curves are shown in Figure 5. Based on these results, we performed multivariate analysis using a Cox proportional hazard model. Among the non-metastatic gastric cancer patients, those who received adjuvant treatment had a HR of 0.5 (95% CI: 0.36 to 0.69), those with PNI had an HR of 1.31 (95% CI: 1.19 to 3.11), those with LVI had an HR of 2.1 (95% CI: 1.1 to 3.7), and those with stage III cancer had an HR of 3.3 (95% CI: 1.67 to 5.89). The details of Cox regression analysis of DFS are shown in Table 4.

**Table 4:** Results of Cox regression analysis of the association between progression-free survival rates, and adjuvant treatment, perineural invasion, lymphovascular invasion, and disease stage in 386 patients with non-metastatic presentation

**Variable**	***P***	**Overall survival**
		HR (95% CI)
**Adjuvant Treatment**	0.02	
No		1
Yes		0.5 (0.36-0.69)
**Perineural invasion**	0.03	
Absent		1
Present		1.31 (1.19-3.11)
**Lymphovascular invasion**	0.011	
Absent		1
Present		2.1 (1.1-3.7)
**Stage**	0.001	
Stage I		1
Stage II		1.6 (1.1 9-1.91)
Stage III		3.3 (1.67-5.89)

CI, confidence interval; HR, hazard ratio.

## Discussion

Gastric cancer is the fourth most commonly diagnosed cancer [13], but the incidence has been declining worldwide [4-6,14,15]. Nonetheless, gastric cancer is the second leading cause of cancer-related mortality in both men and women [13-15]. In fact, gastric cancer rates have increased among older people in both the United States and Japan [9,10]. In the present retrospective study the median age at diagnosis was 58 years. When compared with the Surveillance, Epidemiology, and End Results (SEER) data from 1999 to 2003, the median age in the present cohort was 14 years younger than that of the American patients [7]. In addition, the incidence rate in patients with a median age older than 70 years increased by 14% during the period 1999 to 2002 and by 19.8% between 2007 and 2010. In the present study there was no difference in histopathological features, disease stage, gender, or tumor localization in the stomach between groups 1 and 2.

A Japanese study reported that when compared to patients aged 70 years or younger, more of the patients aged 70 years or older had differentiated histology and fewer had vascular involvement, but there was no difference in disease stage, lymph node involvement, tumor size, lymphatic involvement, or gender [16]. Nonetheless, an earlier study by Arai *et al*.

[17] showed that among patients older than 85 years of age cancer was more often localized in the lower third of the stomach. In contrast to the present study, some Japanese studies observed that the proportion of differentiated-type gastric carcinoma increased with age and histological diversity during growth [16-18], but all of these studies were conducted in the same country and the majority of the patients were of advanced age.

In the present study there were no significant differences in surgical procedures (total *versus* subtotal gastrostomy) between groups 1 and 2; however, a limitation of the present study is that lymph node dissection reports were not available, and, as such, lymph node dissection rates could not be compared. As compared to group 2, fewer of the patients in group 1 with non-metastatic presentation and metastatic presentation received adjuvant treatment and chemotherapy, respectively. Saito *et al*. [16] reported that fewer older patients than patients of other age groups received chemotherapy and lymph node dissection.

Several studies reported that advanced age was associated with shorter survival [16,19,20], whereas others reported that the prognosis in older patients who undergo curative resection is the same as in patients of other age groups [21,22]. In the present study the five-year survival rate in the patients age 70 years or younger with non-metastatic presentation was significantly better than that in the patients who were older than 70 years, and the one-year survival rate in the patients age 70 years or younger with metastatic presentation was significantly higher than in the patients who were older than 70 years. As previous reported [23-26], patients in the present study with non-metastatic presentation, and LVI, PNI, and advanced-stage cancer who underwent curative resection had worse independent prognostic factors for survival, but those who received adjuvant treatment had favorable prognostic factors for survival. In patients older than age 70 years with metastatic presentation and who received only palliative care (no palliative chemotherapy) there was a worse independent effect on survival. Another limitation of the present study is that comorbidity rates were not available and, as such, cancer-specific survival rates in groups 1 and 2 could not be determined.

## Conclusions

The median age at diagnosis and the percentage of elderly patients with gastric cancer increased between 1999 and 2010. Both the elderly and non-elderly patients had similar histopathological features and underwent similar surgical modalities. The elderly patients had lower survival rates than the non-elderly patients, but fewer of them underwent adjuvant treatment and palliative chemotherapy than the non-elderly patients. Patients with PNI and LVI who did not receive adjuvant treatment and who had advanced-stage disease had worse independent prognostic effects on survival than patients with non-metastatic presentation. Lower age and palliative chemotherapy were independent favorable prognostic factors that affected the survival rates in patients with metastatic presentation. The incidence of gastric cancer was higher in the patients who were older than70 years. Additional randomized studies are needed to further assess the safety and clinical benefit of chemotherapy in gastric cancer patients who are older than 70 years of age.

## Abbreviations

CRT: Chemoradiotherapy; DFS: Disease-free survival; HR: Hazard ratio; LVI: Lymphovascular invasion; OS: Overall survival rate; PNI: Perineural invasion; RT: Radiotherapy.

Written informed consent was obtained from the patient for publication of this report and any accompanying images.

## Competing interests

The authors declare that they have no competing interests.

## Authors’ contributions

Analysis and interpretation of the data are realized by all authors. All authors read and approved the final manuscript.
